# Application of through-the-scope twin clip for defect closure after gastric gastrointestinal stromal tumor transoral super minimally invasive surgery resection

**DOI:** 10.1055/a-2346-5017

**Published:** 2024-07-08

**Authors:** Chenyang Li, Shuling Li, Tao Wang, Yan Xu, Zhongrui Xu, Fuxiu Huang, Chao Chen

**Affiliations:** 1 Department of Gastroenterology, The First Medical Center of PLA General Hospital, Beijing, China; 2 Department of Gastroenterology, The Fourth Medical Center of PLA General Hospital, Beijing, China

The closure of large or perforated defects has posed a significant challenge in the evolution of endoscopic treatments. The over-the-scope (OTS) clip, once the mainstay for such closures, has certain limitations, such as the necessity for pre-procedure attachment to the endoscopeʼs tip, which may entail additional surgical entries and potentially longer operative times, increasing the risk of complications like postoperative peritonitis 1 .

We report the application of a through-the-scope twin clip (TTS-TC) for defect closure after submucosal tumor (SMT) dissection, a device initially developed and implemented by Prof. Zhangʼs team 2 . The device features a central support column that enables independent operation of its bilateral clips, with a maximum outer diameter of 2.9 mm, an opening angle of up to 60 degrees, and an opening size of 1.0 cm, making it suitable for effective closure of defects less than 5.0 cm in diameter 3 4 5 .

A 71-year-old man was admitted to our hospital with a 1.8 × 2.0-cm SMT in the gastric antrum. We resected the lesion using super minimally invasive surgery, also known as endoscopic submucosal dissection (ESD). We chose TTS-TCs to close the 3.0 × 4.0-cm post-surgical mucosal defect ( Fig. 1 , Video 1 ). We inserted the TTS-TC through the endoscopic channel and clamped one side of the defect, positioning the other side, and then releasing the TTS-TC. The large defect then transformed into small ones. Subsequently, several traditional through-the-scope clips were deployed to completely close the defect. The patient fasted for 1 day and was discharged after 3 days without any postoperative complications. Histology revealed a gastrointestinal stromal tumor. The TTS-TC emerges as a promising tool for the closure of a large gastric defect, offering a new dimension in endoscopic management.

**Fig. 1:**
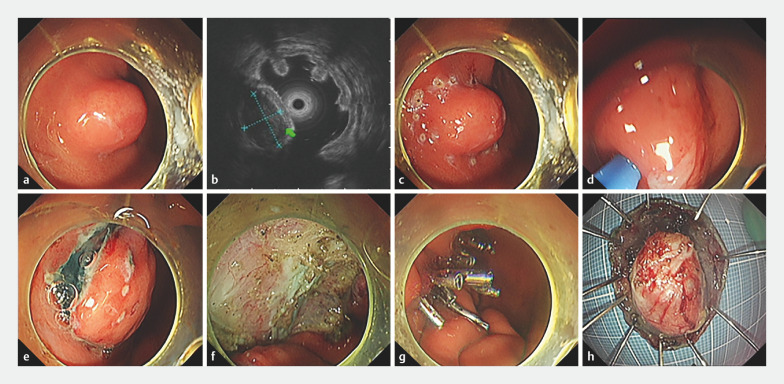
Operation steps of gastric submucosal tumor (SMT) transoral super minimally invasive surgery resection. **a** An SMT was located in the gastric antrum. **b** Endoscopic ultrasound showed that the tumor originated from the muscularis propria. **c** Marking the surrounding mucosa of the SMT. **d** Injection of fluid into the submucosa. **e** The submucosa was dissected. **f** A large defect was formed. **g** Closure of the defect using a through-the-scope twin clip and the traditional clips. **h** The specimen of the SMT.

Closure of a large defect in the gastric antrum with the novel through-the-scope twin clip after super minimally invasive surgery.Video 1

Endoscopy_UCTN_Code_TTT_1AO_2AO

## Notes

Endoscopy E-Videos https://eref.thieme.de/e-videos
*E-Videos* is an open access online section of the journal *Endoscopy* , reporting on interesting cases and new techniques in gastroenterological endoscopy. All papers include a high-quality video and are published with a Creative Commons CC-BY license. Endoscopy E-Videos qualify for HINARI discounts and waivers and eligibility is automatically checked during the submission process. We grant 100% waivers to articles whose corresponding authors are based in Group A countries and 50% waivers to those who are based in Group B countries as classified by Research4Life (see: https://www.research4life.org/access/eligibility/ ). This section has its own submission website at https://mc.manuscriptcentral.com/e-videos .
